# An unusual cause of hemolacria: retained contact lens in upper fornix

**DOI:** 10.22336/rjo.2024.57

**Published:** 2024

**Authors:** Obaidur Rehman, Shreya Keshari, Sima Das

**Affiliations:** Department of Oculoplasty and Ocular Oncology, Dr. Shroff’s Charity Eye Hospital, New Delhi, India

**Keywords:** hemolacria, retained contact lens, upper fornix trap, pyogenic granuloma

## Abstract

A 64-year-old male was referred for complaints of blood in tears and bloody discharge of unknown cause in the left eye. The patient was a chronic bandage contact lens (BCL) user. He had no history of recent trauma. A blood-stained BCL was present on the cornea in the left eye, which was removed. The ocular surface was dry with vascularization of the cornea. Double eversion of the upper eyelid with a Desmarre’s retractor revealed a pyogenic granuloma with large papillae on the forniceal conjunctiva and a folded BCL hidden in the fornix. The folded BCL was carefully removed from the “upper fornix trap” and topical steroid eyedrops were prescribed.

## Background

Retention of rigid and soft contact lenses (CL) in the upper fornix has been well documented in the literature by several authors [1-9]. The “hidden” contact lens may be discovered during routine ophthalmic evaluation or procedures [2,3] or may present as a masquerade of other clinical entities [4-9]. Hemolacria or blood in tears is a disturbing condition for the patient, caused by many etiologies [10]. We describe an unusual presentation of a retained bandage contact lens (BCL) as hemolacria in an elderly male.

## Case presentation

**Fig. 1:**
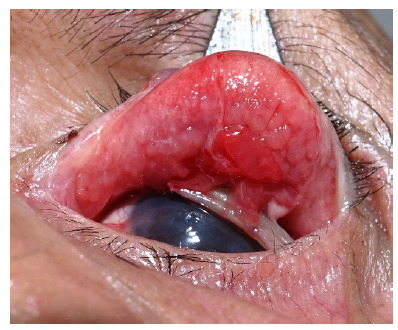
Double eversion of the upper eyelid showing a pyogenic granuloma and papillae on the forniceal conjunctiva with a folded BCL visible below the lesion

A 64-year-old male complaining of blood-stained tears and occasional bloody discharge in his left eye presented to our clinic. The first episode was noted 3 weeks before and the frequency of such episodes had gradually increased. The patient also complained of mild irritation in the left eye. The initial ophthalmic examination at a primary center was reported to be normal and the cause of hemolacria was not identified. No history of recent trauma or ophthalmic surgery was registered. No known systemic disorders, including hypertension or bleeding diathesis, were noted, and no history of epistaxis was recorded. The patient was a chronic user of soft bandage contact lenses, in the left eye, for the past 15 years, following chemical injury to the eye. On ocular examination, blood-stained crusting was noted on the medial eyelashes of the left eye and a blood-stained BCL was present on the left cornea. ROPLAS test was negative and lacrimal syringing was freely patent on the left side. The blood-stained BCL was removed from the corneal surface and a dry corneal surface with superficial vascularization was noted. No other abnormality was observed on the ocular surface, including the lower fornix. The upper tarsal conjunctiva appeared mildly congested. On double eversion of the upper eyelid with a Desmarre’s retractor, a pyogenic granuloma surrounded by large papillae was noted on the forniceal conjunctiva, with a folded BCL hidden in the “upper fornix trap” (**Fig. 1**).

## Results

**Fig. 2:**
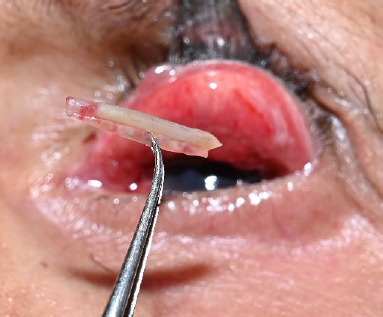
The folded contact lens after removal

After the instillation of topical anesthetic eyedrops, the folded BCL was carefully removed (**Fig. 2**). The patient was prescribed topical Prednisolone acetate 1% eyedrops at 6 hourly intervals. Retrospective history taking revealed frequent episodes of “lost” BCLs over the years, which were assumed to be expelled from the eye by the patient. At the 2-week follow-up, the pyogenic granuloma and papillae had almost resolved, and the patient reported no further episodes of hemolacria since the initial visit.

## Discussion

The onset of hemolacria in the eye is a distressing condition for a patient, due to its dramatic presentation. Hemolacria has a variety of etiologies, including conjunctival and corneal vascular lesions, lacrimal sac tumors, drug application, epistaxis regurgitating through the lacrimal pathway, and malingering [10]. The presentation of hemolacria in the elderly age group is alarming to the clinician and may be perplexing when no obvious cause can be identified. In our patient, while obvious bloodstaining of the eyelashes was present, the ocular surface and lacrimal passage were normal, and no history of epistaxis was recorded.

The “upper fornix trap” was described by Bock [1], who utilized a flattened glass rod to remove a contact lens lodged in the upper cul-de-sac. This trap is formed when the lower edge of the CL gets wedged into the upper border of the tarsal plate, thus, trapping it in the deep upper fornix. Chronic CL users, especially those in the elderly age group may be more prone to retention of “lost” CLs in the upper fornix. Fluorescein staining and sweeping of the upper fornix may reveal the CL hidden from plain sight in such cases. The CL may penetrate surrounding structures and get lodged at various tissue planes, including the subconjunctival plane, the tarsal plate, or the anterior orbit [5]. Granulation tissue around the CL or capsule formation may also occur. Presentation of retained CL as chalazion [4], eyelid swelling [5,9], mass lesion [6], corneal ulcer [7], and chronic conjunctivitis [8] has been described. The cause of hemolacria in our case was the forniceal pyogenic granuloma, which is a lesion known for bleeding spontaneously or on touch.

## Conclusion

Hemolacria due to retained CL in the upper fornix is a unique presentation, which, to our best knowledge, has not been reported previously in the literature. Our case report highlights the significance of meticulous and detailed clinical evaluation, including double eversion of eyelids, especially in patients with a history of lost contact lenses.

## References

[1] Bock RH (1971). The upper fornix trap. Br J Ophthalmol.

[2] Morjaria R, Crombie R, Patel A (2017). Retained contact lenses. BMJ.

[3] Zhao M, Gu T, Teng L, Zhang C (2024). Five Retained Soft Contact Lenses in the Upper Fornix: A Case Report in a Patient with Hemifacial Atrophy. Plast Reconstr Surg Glob Open.

[4] Agarwal PK, Ahmed TY, Diaper CJ (2013). Retained soft contact lens masquerading as a chalazion: a case report. Indian J Ophthalmol.

[5] Shams PN, Beckingsale AB, Sheldrick JH, Rose GE (2011). An unusual eyelid lump: unsuspected embedded contact lens for up to 40 years. Two cases and literature review Eye (Lond).

[6] Rebhun CB, Tran AQ, Belinsky I (2021). Superior fornix mass with retained soft contact lens. Indian J Ophthalmol Case Rep.

[7] Tao BK, Hassanlou M, Ong Tone S (2023). Corneal ulcer as the presenting sign of prolonged contact lens retention over 25 years. Can J Ophthalmol.

[8] Arshad JI, Saud A, White DE, Afshari NA, Sayegh RR (2020). Chronic Conjunctivitis From a Retained Contact Lens. Eye Contact Lens.

[9] Kang H, Takahashi Y, Kakizaki H (2016). Migration of rigid gas permeable contact lens into the upper eyelid after trauma: a case report. BMC Ophthalmol.

[10] Ho VH, Wilson MW, Linder JS, Fleming JC, Haik BG (2004). Bloody tears of unknown cause: case series and review of the literature. Ophthalmic Plast Reconstr Surg.

